# Controlled Biosynthesis of ZnCdS Quantum Dots with Visible-Light-Driven Photocatalytic Hydrogen Production Activity

**DOI:** 10.3390/nano11061357

**Published:** 2021-05-21

**Authors:** Shiyue Qi, Yahui Miao, Ji Chen, Huichao Chu, Bingyang Tian, Borong Wu, Yanju Li, Baoping Xin

**Affiliations:** School of Materials Science and Engineering, Beijing Institute of Technology, Beijing 100081, China; qishiyue@bit.edu.cn (S.Q.); miaoyahui1004@163.com (Y.M.); 18813087655@163.com (J.C.); chuhuichao@bit.edu.cn (H.C.); 3120185571@bit.edu.cn (B.T.); Wubr@bit.edu.cn (B.W.); liyanju@bit.edu.cn (Y.L.)

**Keywords:** biosynthesis, extracellular proteins, sulfate reducing bacteria, photocatalytic hydrogen evolution, ZnCdS quantum dots

## Abstract

The development of visible-light-responsive photocatalysts with high efficiency, stability, and eco-friendly nature is beneficial to the large-scale application of solar hydrogen production. In this work, the production of biosynthetic ternary ZnCdS photocatalysts (Eg = 2.35–2.72 eV) by sulfate-reducing bacteria (SRB) under mild conditions was carried out for the first time. The huge amount of biogenic S^2^^−^ and inherent extracellular proteins (EPs) secreted by SRB are important components of rapid extracellular biosynthesis. The ternary ZnCdS QDs at different molar ratios of Zn^2+^and Cd^2+^ from 15:1 to 1:1 were monodisperse spheres with good crystallinity and average crystallite size of 6.12 nm, independent of the molar ratio of Cd^2+^ to Zn^2+^. All the ZnCdS QDs had remarkable photocatalytic activity and stability for hydrogen evolution under visible light, without noble metal cocatalysts. Especially, ZnCdS QDs at Zn/Cd = 3:1 showed the highest H_2_ production activity of 3.752 mmol·h^−1^·g^−1^. This excellent performance was due to the high absorption of visible light, the high specific surface area, and the lower recombination rate between photoexcited electrons and holes. The adhered inherent EPs on the ZnCdS QDs slowed down the photocorrosion and improved the stability in photocatalytic hydrogen evolution. This study provides a new direction for solar hydrogen production.

## 1. Introduction

The growing demand for energy and concerns about environmental pollution are driving the replacement of materials and equipment for sustainable and renewable energy sources [1]. Hydrogen energy is considered to be one of the most promising clean energy sources because of its high energy density, good recoverability, and lack of pollutant emission [2,3]. Among hydrogen production technologies that have been developed, the solar photolysis of water for hydrogen production is the most environmentally friendly method [4,5]. However, most photocatalysts have poor catalytic performance in visible-light-driven reactions, even if precious metals are used as co-catalysts, which greatly limits their practical application [6,7,8]. Therefore, to solve this problem it is necessary to develop new photocatalysts based on the abundant elements on Earth which can be as efficient as precious-metal-based catalysts.

Among various photocatalysts, metal sulfide quantum dots (QDs) have excellent properties in photocatalytic hydrogen evolution due to their tunable size-dependent electronic properties, quantum confinement effect, and the abundant sulfur on the Earth [9,10,11,12,13,14]. Particularly, CdS QDs have been widely studied in water hydrogen production under visible light, considering their high activity and sufficient negative conduction band position [15]; however, they have high toxicity and poor photocorrosion resistance [16,17]. CdS is toxic to humans, and nanoscale CdS is even more toxic. The toxicity of QDs depends on their size, shape, and surface structure [18]. ZnS is a wide-band-gap material with low toxicity and high photocorrosion resistance for photocatalytic H_2_ evolution, but ZnS only responds to UV light [19]. The lattice constants of zinc sphalerite ZnS and CdS are 5.406 Å and 5.835 Å, respectively. Such a low lattice mismatch makes it easy for Zn to be introduced into the lattice of CdS to form ternary ZnCdS materials, which is a feasible method to improve the band-gap emission efficiency and reduce toxicity [20,21,22], thus covering the shortage of single ZnS and CdS [23]. Since ternary ZnCdS QDs have double boundary-dependent potential, they can prevent photoinduced carrier recombination [24], and have higher catalytic activity for water splitting than binary QDs.

The size of ZnCdS synthesized by traditional methods such as co-precipitation, water/solvothermal method, and calcination is very large (>10 nm) and even leads to the formation of large aggregates [25,26,27,28,29,30]. ZnCdS with particle size of 4.5–7.8 nm can be synthesized by high-boiling-point solvothermal method [31], but it is necessary to control annealing and a sequential sulfidation and ion-exchange procedure by a zeolitic-imidazolate-framework-8 (ZIF-8)-template is required. A large number of expensive materials, such as metal-organic frameworks (MOFs), C_3_N_4_, and reduced graphene oxide (RGO), are needed to obtain ZnCdS quantum dots with small particle size, low aggregation, and high dispersion which have photocatalytic activity and stability [32,33,34,35]. For instance, Zn_0.8_Cd_0.2_S was prepared by a co-precipitation hydrothermal method using RGO as the carrier. Compared with Zn_0.8_Cd_0.2_S without a carrier, the photocatalytic activity was significantly improved [32]. Moreover, using UIO-66-NH_2_ as a dispersant to synthesize Cd_0.2_Zn_0.8_S@UiO-66-NH_2_, the particle size of Cd_0.2_Zn_0.8_S@UiO-66-NH_2_ could be greatly reduced from more than 50 nm to less than 20 nm, and the rate of hydrogen from water splitting was increased by 2.1 times [33]. Core–shell composites are one of the unique types of composite that drastically improve the photo-exciton separation, where chalcogenides in the core can be well protected for sustainable uses [36,37]. The construction of core–shell architecture has many challenges, such as the mono-dispersion of core materials, ensuring intact interface contact with the shell layer, and obtaining uniform wrapping of the core with shell materials.

In general, in order to reduce the particle size of ZnCdS, secondary materials need to be introduced as carriers or dispersants, which prolongs the synthesis process or introduces additional capture centers at the interface to consume photogenerated charge carriers, and reduces the photocatalytic activity. The use of toxic chemicals/solvents; explosive precursors; harsh reaction conditions such as extreme temperature, pressure, and pH; as well as the discharge of harmful by-products has a negative impact on the environment and a high cost [38,39]. Therefore, an eco-friendly and controllable method to fabricate ZnCdS QDs with high uniformity is urgently needed.

Compared with chemical synthesis, biological synthesis follows green chemistry principles, which is more acceptable in terms of environment-friendliness and energy-saving, and can be carried out at ambient temperatures and pressures. Biosynthesis mainly uses microbial cells as microreactors to synthesize inorganic nanocrystals through biomineralization or biological reduction [40]. In the past five years, there have been more than 10 review articles on the biological preparation of metal nanomaterials. In 2015, O.V. Singh led the editing and publication of *Bio-Nanoparticles: Biosynthesis and Sustainable Biotechnological Implications* [41]. In 2020, Choi et al. jointly published an important review of biosynthetic inorganic nanomaterials in *Nature Reviews Chemistry* [40]. In 2021, Saravanan et al. published a review on biosynthetic metal nanomaterials and their environmental applications [42], indicating that the subject of biosynthetic nanomaterials was gradually coming to maturity. A wide range of metal nanoparticles and QDs have therefore been synthesized by diverse microorganisms and biomolecules in the past 20 years [41,43].

There are many research works on the biosynthesis of ZnS and CdS QDs as binary semiconductors, but the biosynthesis of ternary ZnCdS QDs has not yet been reported, due to the difficulty in the stable synthesis of ternary materials in complex biological systems. In previous work, the highest extracellular synthesis rate of ZnS QDS was obtained by mixed sulfate-reducing bacteria (SRB), which secreted extracellular proteins (EPs) containing amino acids to strongly mediate the biosynthesis process of binary metal sulfides QDs [44]. This study provides a simple and efficient method for the extracellular biosynthesis of ZnCdS QDs by SRB for the first time. Importantly, the prepared photocatalysts have activity and photostability in photohydrolysis hydrogen production under visible-light irradiation, aided by a number of adhered EPs, without the use of a noble metal cocatalyst. This EPs-mediated efficient biosynthesis method can open a new perspective for the synthesis of various highly active ZnCdS QDs.

## 2. Materials and Methods

### 2.1. Chemicals, Microorganisms and Media

All analytical chemical reagents were obtained from Beijing Chemical Company (Beijing, China) and used as received without further purification, including sodium lactate as an electron donor, Na_2_SO_4_ (SO_4_^2−^) as an electron acceptor, and ZnSO_4_/CdCl_2_ as precursor Zn^2+^/Cd^2+^ for the biosynthesis of ZnCdS QDs. Deionized water was used to prepare the solutions for both cell culture and the biosynthesis of ZnCdS QDs.

In previous studies, we domesticated, screened, and isolated mixed SRB from sulphate-rich soil consisting of 25% *Desulfovibrio* sp., 25% *Clostridiaceae* sp., 25% *Proteiniphilum* sp., 12.5% *Geotoga* sp., and 12.5% *Sphaerochaeta* sp. The SRB, which had harvested the highest-ever extracellular biosynthesis of binary metal sulfide QDs [44], were applied to fabricate the ternary ZnCdS QDs in a modified, easier, and faster manner in the current work. The growth medium contained 0.1 g/L CaCl_2_, 1.0 g/L NH_4_Cl, 0.5 g/L K_2_HPO_4_, 0.5 g/L MgSO_4_, 0.1 mol/L sodium lactate, and 0.1 mol/L Na_2_SO_4_, at pH 7.2. The mixed SRB were inoculated in fresh medium at 10% (*v*/*v*) density and then anaerobically cultured at 35 °C.

### 2.2. Biosynthesis of ZnCdS Photocatalysts

When the SRB reached logarithmic phase after about 4 days of culture, the turbidity of SRB (OD_600_) increased from 0.1 to 0.6. At that time, the cells were removed by centrifuging at 8000 rpm for 10 min to obtain the supernatant, which contained a massive amount of biogenic H_2_S (S^2−^) from sulfate bio-reduction as well as specific EPs secreted by the SRB [44]. Subsequently, 200 mL of the SRB-derived active supernatant was mixed with 200 mL of mixed solutions of ZnSO_4_ and CdCl_2_ with different molar ratios (Zn:Cd = 0:1, 1:1, 3:1, 5:1, 9:1, 15:1, 1:0; total concentration of Zn^2+^ and Cd^2+^ = 0.1 mol/L). The EPs-mediated precipitation reaction between biogenic S^2^^−^ and the mixed solution of Zn^2+^ and Cd^2+^ occurred in a 500 mL beaker with a magnetic stirrer (30 rpm, 25 °C). After 30 min of contact, the colorless transparent solution gradually changed into a milky white or slightly milky yellow suspension, displaying the formation of alloyed ZnCdS QDs. The precipitates were collected by centrifugation (8000 rpm, 10 min) and washed with deionized water and ethanol 3 times. Finally, the samples were sealed and frozen at −80 °C for 12 h, and then the samples were unsealed at −50 °C for vacuum drying and ground with an agate mortar into a powder for use, so as to keep the sample morphology unchanged.

### 2.3. Characterization of the Extracellular Biosynthesized ZnCdS QDs

The crystal phases of vacuum-dried ZnCdS powders were characterized on an X-ray diffractometer (XRD, Rigaku RINT-2000, Toyko, Japan) using Cu Kα radiation at a scan rate of 8°/min. The morphology and size of the materials were obtained by transmission electron microscopy (TEM, FEI Tecnai TF20, Waltham, MA, USA) at an acceleration voltage of 200 kV. Ten TEM images were randomly selected, and the Nano Measurer 1.2 software was used to calculate the sizes of 100 single particles in the images, and to analyze the size distribution and average crystallite size (ACS). The purities and compositions of the ternary QDs were investigated using energy-dispersive X-ray spectroscopy (EDS, Oxford, UK) with an instrument operating at 20 kV. The surface functional groups of ZnCdS QDs were analyzed by Fourier transform infrared spectroscopy (FTIR, Thermo Nicolet iS10, Waltham, MA, USA). The thick film of the analytes was prepared by the mixture of KBr and the sample. Ultraviolet–visible diffuse reflectance spectra (UV–vis DRS, UV-2550PC, Shimadzu, Japan) were measured using a UV-2550 (Shimadzu, Japan) spectrometer. Photoluminescence (PL) spectra were afforded at the excitation wavelength of 350 nm performed on a Hitachi F-4500 fluorescence spectrometer (Tokyo, Japan). According to standard procedures (ISO 13903-2005), the amino acid types and concentrations in the proteins that mediated the synthesis of ZnCdS QDs were analyzed using an amino acid analyzer (L-8900, Hitachi High-Tech, Toyko, Japan) [44].

### 2.4. Photocatalytic Hydrogen Production of ZnCdS QDs

Photocatalytic water decomposition reaction was carried out in a photocatalytic hydrogen evolution system (LabSolar-6A, Perfectlight, Beijing, China). Twenty-five milligrams of the biosynthesized photocatalyst was suspended in 50 mL of a 0.75 M Na_2_S/1.05 M Na_2_SO_3_ solution and sealed under ultrasonic vibration for 20 min. Before the irradiation, the system was bubbled with nitrogen for 30 min to completely remove the dissolved oxygen and create an anaerobic atmosphere. A 300 W Xe lamp (Perfect Light, Microsolar 300, Beijing, China) equipped with a UV cut-off filter (λ > 420 nm) served as a light source for photocatalytic hydrogen production. A gas chromatograph (TechComp, GC 7900, Tianmei Co., Ltd, Shanghai, China), a thermal conductivity detector, and high-purity Ar carrier gas were used to detect the photocatalytic H_2_ evolution rate. For each assessment of hydrogen production, 0.5 mL of the headspace was injected into the GC and was quantified with a calibration chart of an external hydrogen standard. Our preliminary experiments demonstrated that no hydrogen was detected in the absence of light irradiation or photocatalysts, suggesting that the hydrogen production was essentially from a photocatalytic reaction.

After the photocatalytic reaction was tested for 5 h, Na_2_S and Na_2_SO_3_ were added to the reaction system, then oxygen was removed and the cycle was begun. It was repeated four times under the same conditions as the first reaction.

## 3. Results

### 3.1. Phase Analysis of the Resulting Products

**Figure 1:**
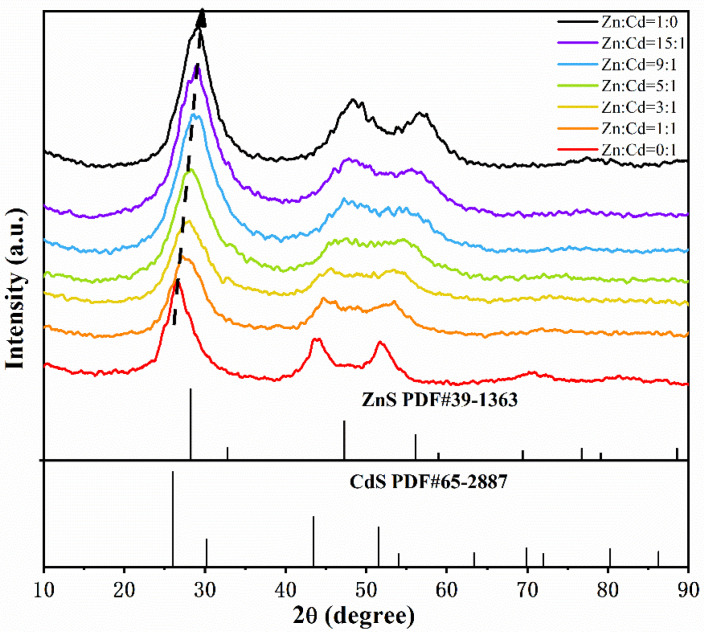
XRD spectra of the resulting products biosynthesized by the SRB-derived supernatants at different molar ratios of Zn^2+^ and Cd^2+^ from 15:1 to 1:1 (Zn/Cd = 0:1 and Zn/Cd = 1:0 as controls for CdS and ZnS). The two sets of lines on the *x*-axis (JCPDS No. 39-1363 and JCPDS No. 65-2887) are the standard spectra for ZnS and CdS, respectively.

The XRD images show that the three major characteristic diffraction peaks are attributable to lattice planes (111), (220), and (311) of the as-prepared products from Zn^2+^/Cd^2+^ = 0:1 and Zn^2+^/Cd^2+^ = 1:0 are well indexed to the standard diffraction patterns of CdS (JCPDS#65-2887) and ZnS (JCPDS#39-1363), respectively (Figure 1). This indicates that the SRB-derived supernatant is capable of biosynthesizing both CdS and ZnS as controls. The resulting products from different molar ratios of Zn^2+^ to Cd^2+^ from 15:1 to 1:1 also had three major characteristic diffraction peaks, respectively attributed to lattice planes (111), (220), and (311), indicating the rapid synthesis of ternary ZnCdS by the SRB-derived supernatant. Compared with ZnS and CdS, the XRD peak position of Zn_1−x_Cd_x_S has a slight shift on the (111) crystal plane to lower diffraction angles (2θ) from 29.1° to 28.7°, 28.2°, 27.9° and 27.5°, respectively, when the concentrations of Cd were gradually increased (Figure 1). No impurity peaks are present in the XRD patterns of ZnCdS, indicating that the biosynthesized ZnCdS crystal had high purity. As a result, it can be said that the similar structure and small difference of bond length between ZnS and CdS are favorable for the biosynthesis of ZnCdS [45].

### 3.2. Shape and Size of the As-Prepared ZnCdS

**Figure 2:**
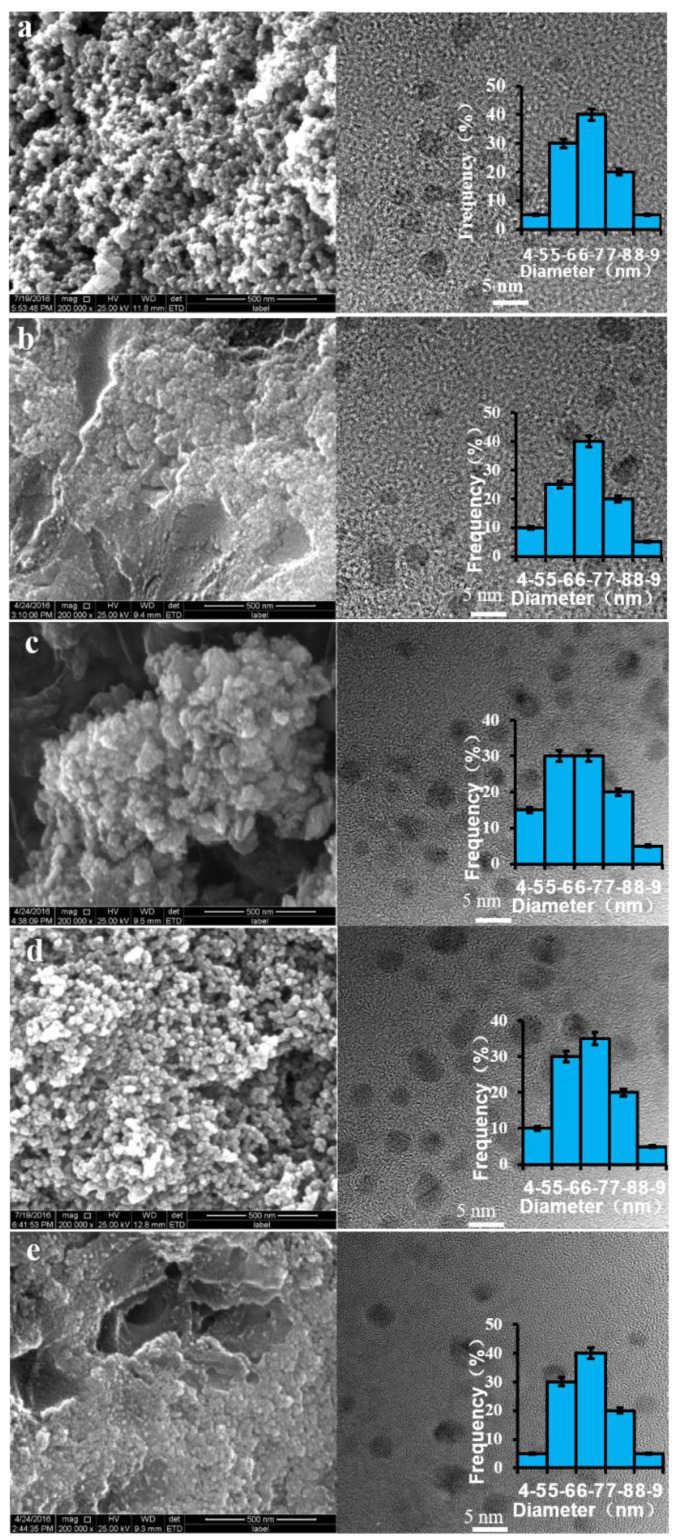
STEM and HRTEM photographs and the average crystallite size for the SRB-derived ternary ZnCdS alloyed QDs at different molar ratios of Zn/Cd: 1:1 (**a**), 3:1 (**b**), 5:1 (**c**), 9:1 (**d**), and 15:1 (**e**).

**Figure 3:**
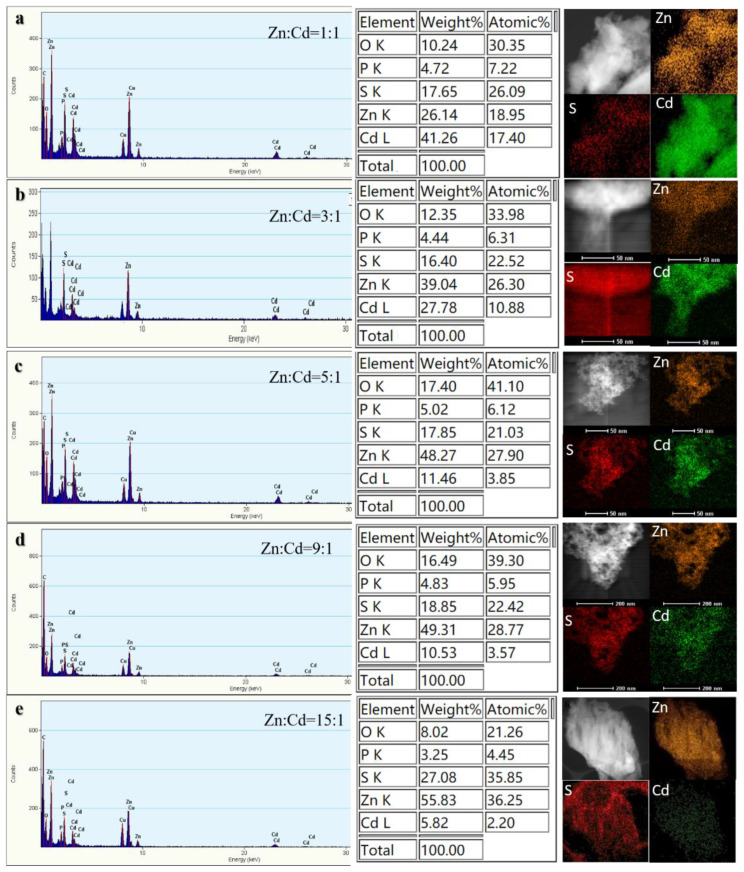
EDX patterns for the SRB-derived ternary ZnCdS alloyed QDs at different molar ratios of Zn/Cd: 1:1 (**a**), 3:1 (**b**), 5:1 (**c**), 9:1 (**d**), and 15:1 (**e**).

The biosynthesized ternary ZnCdS was analyzed using TEM to accurately understand their structure, morphology, and size (Figure 2). The HRTEM photographs reveal that all the as-prepared ZnCdS samples with different molar ratios of Zn^2+^ to Cd^2+^ were monodisperse, well-distributed, fairly uniform spheres. The average crystallite size (ACS) of the as-prepared ZnCdS was 6.12 nm, suggesting that the SRB-derived supernatant rapidly fabricated ZnCdS QDs, independent of the molar ratio of Cd^2+^ to Zn^2+^. The EDX images further show that the actual molar ratios of Zn^2+^ to Cd^2+^ in the biosynthesized ZnCdS were very close to those in the mixed solutions of Zn^2+^ and Cd^2+^, and that total molar concentrations of Zn^2+^ and Cd^2+^ were almost equal to that of the sulfur moiety (S^2−^), accounting for the high purity of the crystal materials (Figure 3). Elements C, O, and P were detected because of the existence of EPs which adhered to the as-prepared products.

### 3.3. EPs’ Roles in Mediating ZnCdS QDs Synthesis

**Figure 4:**
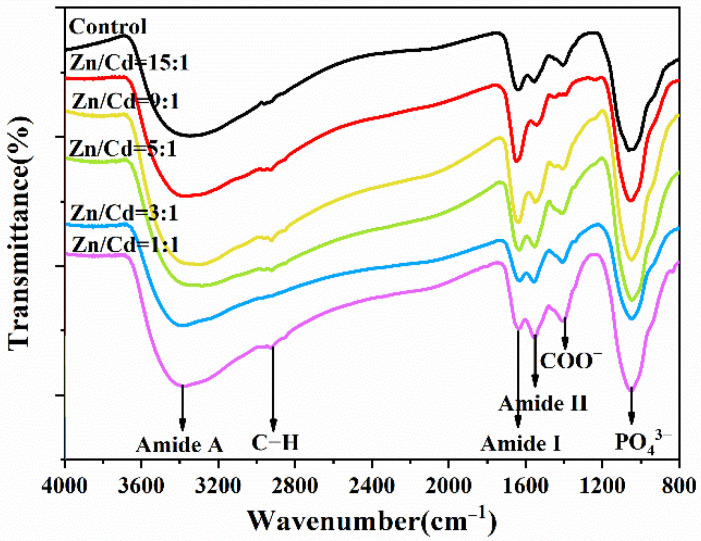
FTIR spectra of the ZnCdS QDs under different molar ratios of Zn/Cd ranging from 15:1 to 1:1. The control was EPs without ZnCdS QDs.

**Figure 5:**
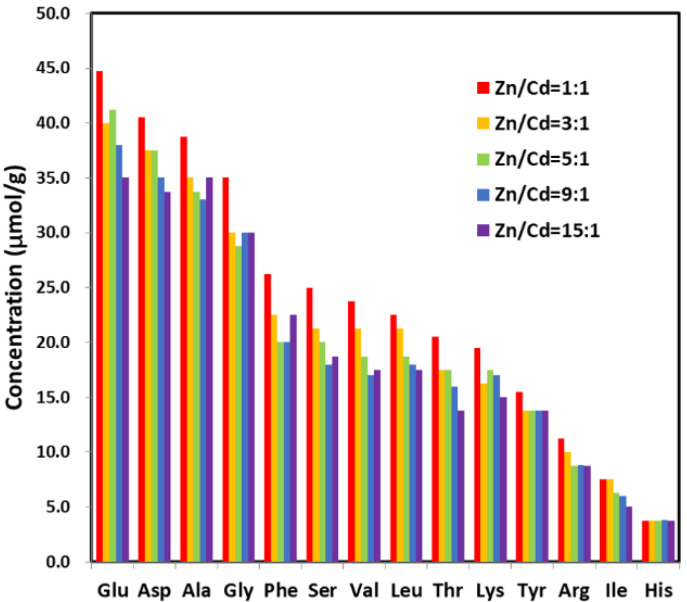
The amino acid species of the adhered EPs and their contents (µmol/g) in the as-prepared ZnCdS QDs under different molar ratios of Zn/Cd ranging from 15:1 to 1:1.

The functional groups in the surface of ZnCdS QDs were analyzed using FTIR to explore the interactions between the ZnCdS QDs and SRB-derived biomolecules during biosynthesis (Figure 4). The control was EPs without ZnCdS QDs. It was clear that the SRB-derived ZnCdS QDs contained abundant functional groups of amides (3293 cm^−1^, 1651 cm^−1^, and 1541 cm^−1^), C–H (2927 cm^−1^), COOH (1405 cm^−1^), and PO_4_^3−^ (1065 cm^−1^) (Figure 4), which showed distinct signals of protein functional groups, just like the control in [46], suggesting that the rapid in vitro synthesis of ZnCdS QDs is mediated by EPs. A further analysis was carried out to explore the species and amino acid contents of the QDs-adhered EPs with different molar ratios of Zn^2+^/Cd^2+^ (Figure 5). It was found that the biosynthesized ZnCdS QDs from different molar ratios of Zn^2+^/Cd^2+^ had almost identical amino acid compositions and contents, demonstrating that the rapid in vitro biosynthesis of ZnCdS QDs under different molar ratios of Zn^2+^/Cd^2+^ is mediated by the same EPs. With increasing Cd^2+^ content, an increasing amount of EPs were adhered to the QDs. The mediating EPs had high total concentrations of acidic amino acids (Glu and Asp) and nonpolar amino acids (Ala, Phe, Val, Leu, Ile). As the first step in the rapid biosynthesis of ZnCdS QDs, negatively charged acidic amino acids provide a large number of adsorption sites for metal ions. A large number of non-polar amino acids make EPs form hydrophobic bridging and smaller cavities, thus controlling the growth of ZnCdS crystals [44].

### 3.4. Optical Properties of ZnCdS QDs

**Figure 6:**
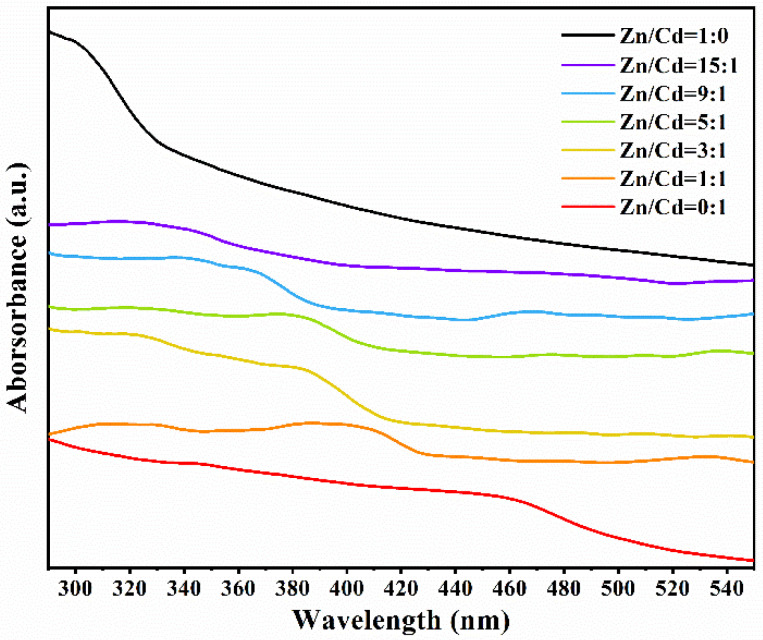
The UV–vis diffuse reflection absorption spectra of the SRB-derived ternary ZnCdS QDs at different molar ratios of Zn^2+^ and Cd^2+^ from 15:1 to 1:1.

In order to detect the light absorption capacity and band-gap energy of ZnCdS quantum dots, UV–vis diffuse reflection absorption spectra were obtained (Figure 6). All ZnCdS QDs exhibited strong absorption bands, indicating that the absorption came from an internal band-gap transition from the valence band to the conduction band. The absorption peaks of ZnCdS QDs are located between the absorption peaks of CdS and ZnS. With the increase of Zn content, the absorption peaks of quantum dots gradually shifted to a shorter wavelength, which also indicates that the ZnCdS was an alloy compound rather than a simple physical mixture of CdS and ZnS. The results of UV–vis diffuse reflection absorption are also consistent with the continuous shift of the XRD diffraction peaks to a higher angle as shown in Figure 1.

**Figure 7:**
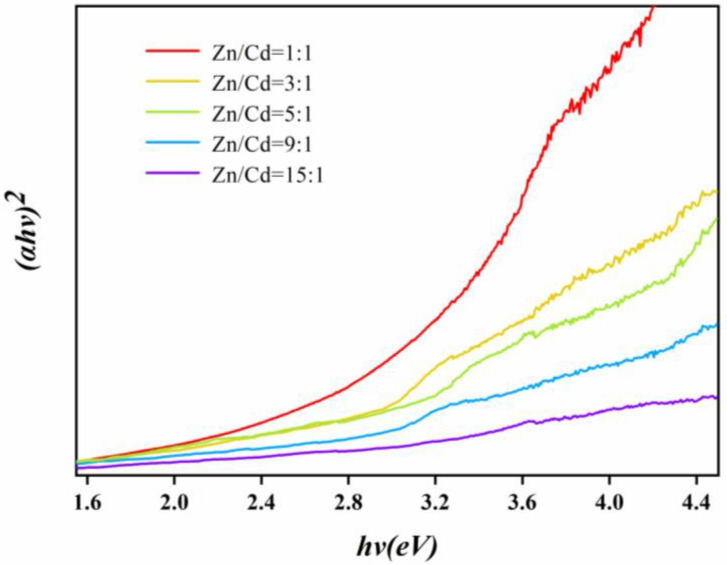
Plots of (*αhν*)^2^ versus band-gap energy (*hν*) of the SRB-derived ternary ZnCdS QDs at different molar ratios of Zn^2+^ and Cd^2+^ from 15:1 to 1:1.

Calculation of the band-gap width of detected materials by the absorption of UV–visible diffuse reflection spectrum has been widely used in photocatalytic studies [47]. According to the Kubelka–Munk formula, the band-gap energies of the ZnCdS bio-QDs could be estimated by the Tauc plot:

(*αhν*)^2^ = A(*hν* − *Eg*) (1)

where *α* is an absorption coefficient, *hν* is photon energy, and A is a constant. The band-gap energies (*Eg*) can be taken as the intercept of the linear part of Tauc plot (*αhν*)^2^ = 0 (Figure 7). The band gaps of alloyed ZnCdS could be adjusted between 2.35 and 2.72 eV. These results indicate that the band gaps of the ZnCdS QDs can be accurately regulated by changing the molar ratios of Zn/Cd precursors.

**Figure 8:**
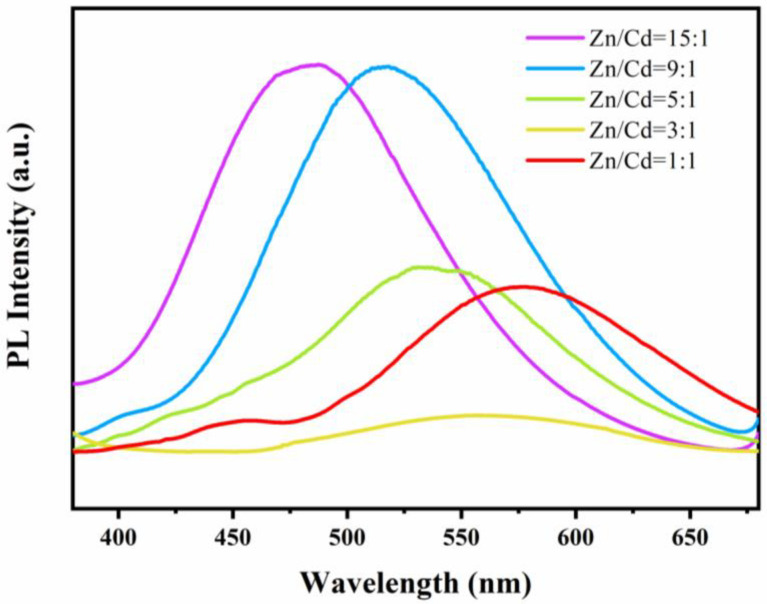
Photoluminescence spectra of the SRB-derived ternary ZnCdS alloyed QDs at different molar ratios of Zn^2+^and Cd^2+^ from 15:1 to 1:1. The image of the QDs under 350 nm UV light.

In general, the combination of photogenerated electron pairs can release energy to induce photoluminescence (PL) emission. Therefore, in order to investigate the photogenerated electron–hole pair separation efficiency of the SRB-derived ternary ZnCdS QDs and prove the excellent charge transfer performance, the PL emission spectra of these samples were examined at an excitation wavelength of 350 nm (Figure 8). All the ZnCdS QDs with different molar ratios had a wide fluorescence emission peak in the visible region. As the molar ratio of Zn^2+^ to Cd^2+^ increased from 1:1 to 15:1, the PL emission peak of ZnCdS QDs shifted from 600 nm to 460 nm, which is a Stokes shift caused by S vacancies [48]. Additionally, the fluorescence intensity of ZnCdS at Zn/Cd = 15:1 was much higher than that of ZnCdS at other molar ratios, indicating that many more electron–hole pairs of ZnCdS at Zn/Cd = 15:1 were generated under irradiation [49]. The emission intensity of ZnCdS was the lowest when Zn/Cd = 3:1, which indicates that the electron–hole recombination of this catalyst was the lowest. The PL intensity of ZnCdS followed the order Zn/Cd = 3:1 < Zn/Cd = 1:1 < Zn/Cd = 5:1 < Zn/Cd = 9:1 < Zn/Cd = 15:1, which is consistent with the law that the lower the recombination rate of photogenerated electrons and holes, the higher the photocatalytic activity. When Zn/Cd = 3:1, the fluorescence of ZnCdS QDs was almost quenched, suggesting that the inhibition of photoexcited electron and hole complex was the most effective and the photocatalytic activity was the highest. Similar results have also been previously reported. Yingwei Li reported the rational design of a new kind of visible-light photocatalyst, hollow ZnCdS rhombic dodecahedral cages, fabricated via simple sulfurization and cation exchange using zeolitic-imidazolate-framework-8 (ZIF-8) as the single precursor [31]. Zn_0.6_Cd_0.4_S showed the highest catalytic activity, with an average H_2_ production rate of 5.68 mmol·h^−1^·g^−1^, ranking among the highest for reported ZnCdS photocatalysts under cocatalyst-free and visible light irradiation (l > 420 nm) conditions. The PL of Zn_0.6_Cd_0.4_S was almost quenched, indicating the most efficient suppression of the recombination between photoexcited electrons and holes, as well as the highest photocatalytic activity.

### 3.5. Photocatalytic Hydrogen Evolution Activity of ZnCdS QDs 

**Figure 9:**
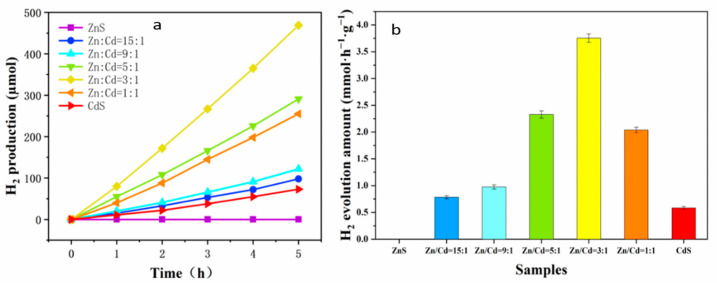
(**a**) Time-dependent photocatalytic H_2_ evolution and (**b**) average hydrogen production rates of the ZnCdS samples.

In the absence of any co-catalysts, the photocatalytic activities of H_2_ production over ZnCdS QDs were detected under visible light irradiation (λ > 420 nm) in an aqueous solution containing S^2−^ and SO_3_^2−^ ions. As shown in Figure 8, the photocatalytic H_2_ production activity varied with the composition of ZnCdS QDs. All ZnCdS QDs had higher photocatalytic hydrogen production performance than those of bulk CdS and ZnS. Since ZnS is only active under ultraviolet light, ZnS has no photocatalytic H_2_ production activity under visible light [18]. The poor photocatalytic hydrogen production activity of single CdS (0.584 mmol·h^−1^·g^−1^) was attributed to the rapid recombination of photogenerated electron–hole pairs. QDs with higher specific surface area have more exposed active sites, facilitating electron–hole separation and transportation [50]. The photocatalytic activity of ZnCdS at Zn/Cd = 3:1 was the best, and the total hydrogen production on 25 mg photocatalyst is 469 μmol after 5 h reaction (Figure 9a). As shown in Figure 9b, the average hydrogen production rate increased first and then decreased with the increase of Cd content (obtained by three repeated tests). In particular, the corresponding H_2_ production rates were 0.784, 0.976, 2.328, 3.752, and 2.040 mmol·h^−1^·g^−1^ for the ZnCdS at Zn/Cd = 15:1, 9:1, 5:1, 3:1, and 1:1, respectively. The photocatalytic activity of ZnCdS QDS was significantly higher than that of ZnS QDs, which should be attributed to the strong visible light absorption of the catalyst, as shown in the UV–vis results (Figure 6). The higher the recombination rate between photoexcited electrons and holes, the lower the photocatalytic activity [31]. As expected, ZnCdS at Zn/Cd = 3:1 exhibited the highest hydrogen production rate. Compared with other ZnCdS QDs, the PL peak intensity of ZnCdS QDs was greatly inhibited when Zn/Cd = 3:1, indicating that the photoinduced carrier recombination was effectively suppressed, thus improving the performance of photocatalytic hydrogen production [50]. The experimental results show that the photocatalytic performance of ZnCdS QDs could be optimized by adjusting the molar ratio of Zn/Cd.

**Figure 10:**
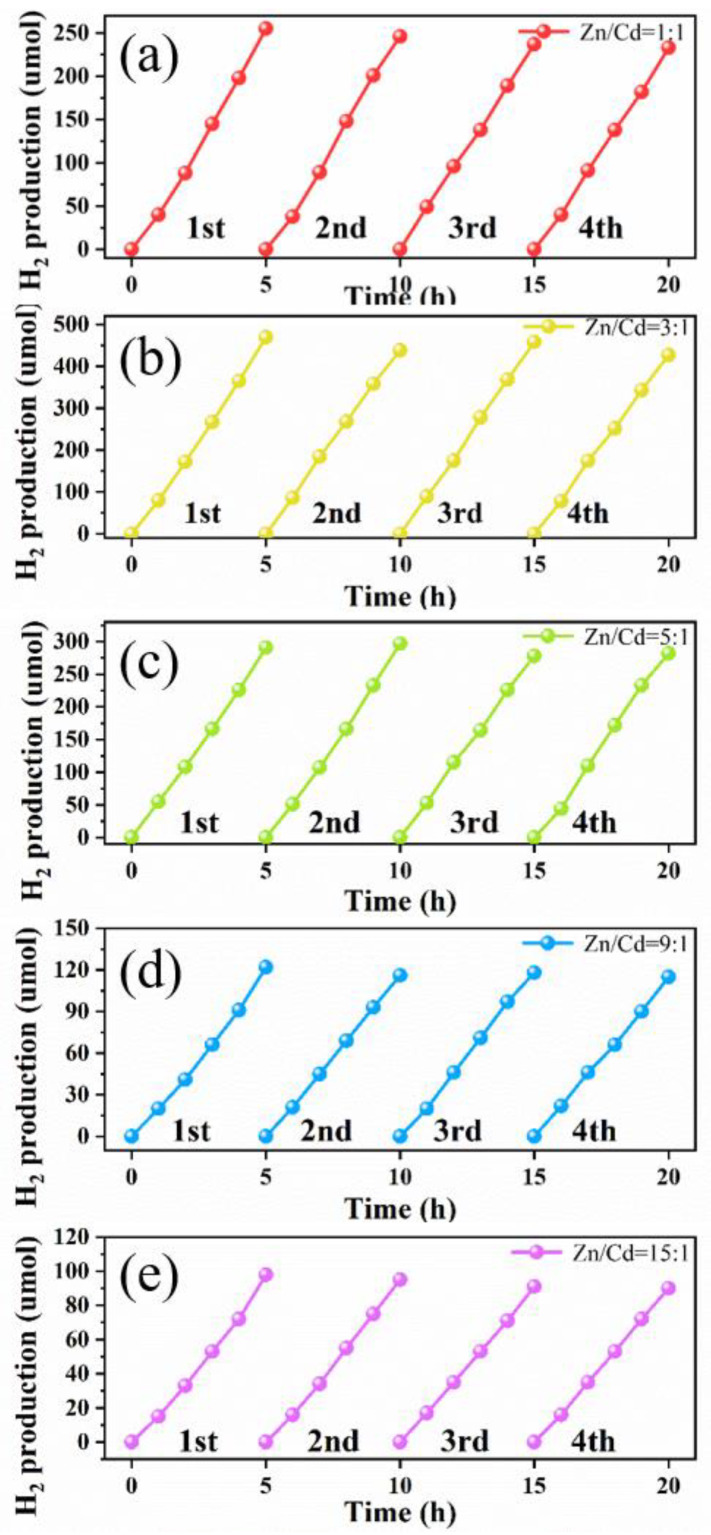
Recyclability of ZnCdS QDs at different molar ratios of Zn/Cd: 1:1 (**a**), 3:1 (**b**), 5:1 (**c**), 9:1 (**d**), and 15:1 (**e**) in photocatalytic hydrogen generation in four consecutive runs.

In addition to the catalytic activity of photocatalysts, the stability of a photocatalyst is another important index to determine whether it can be widely used. The stability of the ZnCdS photocatalysts were tested by a hydrogen evolution cycle of four successive reactions (i.e., a total of 20 h irradiation). As displayed in Figure 10, after four consecutive cycles of experiments, the rate of H_2_ production did not decrease significantly and all photocatalysts maintained 90% of their initial catalytic activity, which shows the high stability and persistence of ZnCdS QDs at different molar ratios of Zn^2+^ and Cd^2+^ from 15:1 to 1:1. The high stability of the biosynthesized ZnCdS QDs is possibly attributable to the negatively charged acidic amino acids electrostatically attracted to Zn^2+^/Cd^2+^, leading to very firm adhesion of EPs to ZnCdS QDs to form solid aggregations. The EPs coating plays a protective role on the QDs and slows down the photocorrosion [45].

## 4. Discussion

Before the photocatalytic reaction, grinding and ultrasonic vibration were used to make the photocatalysts more evenly dispersed without changing the size of the samples. The anaerobic environment made the photocatalytic reaction free of oxygen interference. The choice of sacrificial agent mainly depends on the physical and chemical properties of photocatalyst and the redox potential of sacrificial agent.When the photocatalytic reaction used Na_2_S/Na_2_SO_3_ as a sacrificial agent for sulfide photocatalysts, the main production is sodium sulfate, which will not damage the photocatalyst.

Meiying Liu and colleagues reported an L-cystine-assisted hydrothermal route at moderate temperature to prepare nanosized Zn_x_Cd_1−x_S solid solutions [45]. Like the extracellular proteins coated on the surface of ZnCdS in this study, L-cystine was used as the coordinating agent to control the synthesis. Through adjusting the molar ratios of the precursors, the compositions, sizes, shapes, and properties of Zn_x_Cd_1−x_S solid solutions could be controlled. The research content of the above study is similar to that of this study, so it can be used as a control for the classical chemical methods in this study. Cocatalyst-free Zn_0.5_Cd_0.5_S showed a super high H_2_ production activity of 18.3 mmol·h^−1^·g^−1^. The unusual photocatalytic activity can be attributed to the strong absorption of visible light photons and appropriate edge potential of conduction and valence bands along with the highly efficient charge transport due to the formation of alloyed ZnCdS solid solution. L-cystine, as an organic S source, is prone to be dissolved into water medium under strong alkali condition (pH = 10–11). The crystallization of catalyst in the water medium in hydrothermal conditions over a prolonged period is beneficial to photocatalytic water reduction under visible light irradiation. Under high temperature and high pressure alkaline conditions, the surface composition of the photocatalyst was homogeneous, so its photocatalytic activity was higher than that of the present study.

The specific surface area test results (not shown) indicated that the specific surface area of each sample had little difference. Combined with the TEM images and particle size distribution diagrams in Figure 2, it can be seen that the photocatalysts were zero-dimensional nanometric materials, and their morphologies and structures were similar. Therefore, the difference in specific surface area was not the reason for the change of photocatalytic hydrogen production rate [48].

In reference [48], ZnCdS nanoflowers were prepared by a one-pot hydrothermal method, and the highest H_2_ production activity was 12.57 mmol·h^−1^·g^−1^. In a different study, hollow ZnCdS rhombic dodecahedral cages were fabricated via simple sulfurization and cation exchange using zeolitic-imidazolate-framework-8 (ZIF-8) as the single precursor, and the highest H_2_ evolution rate was 5.68 mmol·h^−1^·g^−1^ [31]. Chen et al. designed ZnCdS QDs by controlled annealing and sequential sulfidation and ion-exchange procedure with a zeolitic-imidazolate-framework-8 (ZIF-8) template, and the highest H_2_ production activity was 3.70 mmol·h^−1^·g^−1^ [50]. In this study, the highest hydrogen production rate was 3.752 mmol·h^−1^·g^−1^. Although the hydrogen production rate is similar to that of ZnCdS quantum dots reported in the literature and is not outstanding among ZnCdS with various morphologies and structures, it is based on a synthesis method involving microorganisms under normal temperature and pressure conditions. This work represents a significant advancement in the green synthesis of metal chalcogenides, which can be used directly (cocatalyst-free) in the natural environment of heavy metal waste water for highly efficient solar H_2_ production.

## 5. Conclusions

This is the first report on the biosynthesis of visible-light-responsive ZnCdS photocatalysts by extracellular biosynthesis. The SRB-derived active supernatant, which contains a massive quantity of biogenic S^2−^ and a high content of specific EPs, enables the rapid and extracellular biosynthesis, which lays the foundation for large-scale production. The as-prepared ZnCdS QDs at different molar ratios of Zn^2+^and Cd^2+^ from 15:1 to 1:1 had good crystallinity, and were well-distributed monodisperse spheres with an ACS of 6.12 nm, independent of the molar ratio of Cd^2+^ to Zn^2+^. All the as-obtained ZnCdS QDs showed excellent activity and stability in photocatalytic hydrogen production. Especially, ZnCdS QDs at Zn/Cd = 3:1 showed the highest H_2_ production activity of 3.752 mmol·h^−1^·g^−1^, exceeding the activity of CdS. The higher photocatalytic activity of ZnCdS QDs is due to its strong absorption of visible light, high specific surface area of exposed active sites, and the lower the recombination rate of photoexcited electrons and holes. The electrostatic-force-driven firm links between the ZnCdS QDs and the specific EPs slows down the photocorrosion, and the amount of adhered EPs on the ZnCdS QDs improve their stability in photocatalytic hydrogen evolution. The multiple functions of the EPs are unique in the biosynthesized visible-light-responsive ZnCdS photocatalysts. In this study, microbial synthesis technology was introduced to produce a ternary metal sulfide photocatalyst to provide a new perspective for effectively improving the stability and durability of solar hydrogen production.

## Data Availability

The data presented in this study are available on request from the corresponding author.
